# Ependymal cell contribution to scar formation after spinal cord injury is minimal, local and dependent on direct ependymal injury

**DOI:** 10.1038/srep41122

**Published:** 2017-01-24

**Authors:** Yilong Ren, Yan Ao, Timothy M. O’Shea, Joshua E. Burda, Alexander M. Bernstein, Andrew J. Brumm, Nagendran Muthusamy, H. Troy Ghashghaei, S. Thomas Carmichael, Liming Cheng, Michael V. Sofroniew

**Affiliations:** 1 Divison of Spine Surgery, Department of Orthopaedics, Tongji Hospital, Tongji University School of Medicine, Shanghai 200065, China; 2 Department of Neurobiology, David Geffen School of Medicine, University of California, Los Angeles, CA 90095, USA; 3 Department of Neurology, David Geffen School of Medicine, University of California, Los Angeles, CA 90095, USA; 4 Department of Molecular Biomedical Sciences, College of Veterinary Medicine, North Carolina State University, Raleigh, NC 27607, USA

## Abstract

Ependyma have been proposed as adult neural stem cells that provide the majority of newly proliferated scar-forming astrocytes that protect tissue and function after spinal cord injury (SCI). This proposal was based on small, midline stab SCI. Here, we tested the generality of this proposal by using a genetic knock-in cell fate mapping strategy in different murine SCI models. After large crush injuries across the entire spinal cord, ependyma-derived progeny remained local, did not migrate and contributed few cells of any kind and less than 2%, if any, of the total newly proliferated and molecularly confirmed scar-forming astrocytes. Stab injuries that were near to but did not directly damage ependyma, contained no ependyma-derived cells. Our findings show that ependymal contribution of progeny after SCI is minimal, local and dependent on direct ependymal injury, indicating that ependyma are not a major source of endogenous neural stem cells or neuroprotective astrocytes after SCI.

Generating newly proliferated cells after tissue injury is a critical adaptation that limits damage, replaces lost tissue and sustains organ function^1^. In the central nervous system (CNS), this proliferative response produces new neural and non-neural cells^2^. Understanding the lineage derivation of injury induced new neural cells may help to identify cell sources that can be manipulated or grafted to improve functional outcome^2^,^3^,^4^,^5^.

After CNS injury and disease, newly proliferated reactive astrocytes form glia-limitans-like scar borders around damaged tissue^6^,^7^,^8^. Transgenic loss-of-function manipulations indicate critical neuroprotective functions of newly proliferated and reactive astrocytes after traumatic injury to brain^9^,^10^,^11^ or spinal cord^12^,^13^, autoimmune disease^8^,^14^,^15^, stroke^16^, infection^17^, and various neurodegenerative diseases^18^,^19^. Moreover, newly proliferated scar-forming astrocytes can support appropriately stimulated axon regeneration^20^. Such observations have led to increasing interest in the origin and lineage derivation of newly proliferated astrocytes generated after CNS damage.

Cell lineage tracing can be conducted *in vivo* in adult transgenic mice by using inducible genetic recombination technology in which tamoxifen dependent Cre-recombinase (CreERT) activates reporter gene expression targeted by specific promoters^21^. This technology can fate map the contribution of specific cell types present in uninjured tissue to newly proliferated cells generated after injury. Using such technology with Nestin-CreERT or human FOXJ1-CreERT promoters driving CreERT expression, ependymal cell progenitors have prominently been proposed as a major population of adult neural stem cells that give rise to migrating progeny that spread to form the majority of the newly-proliferated scar forming astrocytes that restrict tissue damage and protect against neuronal loss after spinal cord injury (SCI)^22^,^23^,^24^,^25^. These broad interpretations were extrapolated from lineage analyses conducted using a highly specialized SCI model of radially penetrating stab injuries placed longitudinally along the spinal cord midline. In contrast, using the same Nestin-Cre-ERT-reporter mice, few ependymal-derived cells were observed in lesions after a full transverse crush SCI and few of these were astrocytes^26^. Although quantification was not conducted, these findings suggested that contrary to previous reports, ependymal contribution to newly proliferated astrocytes might not be a broad feature of more common SCI models that involve damage to larger areas of tissue.

Our laboratory has a longstanding interest in understanding the roles of scar-forming and reactive astrocytes in CNS injury and disease^6^,^10^,^12^,^13^,^20^,^27^. This interest extends to investigating ways in which astroglia might be manipulated or grafted to repopulate the often large areas of non-neural lesion cores that persist after traumatic injury or stroke, as a step towards improving outcome^2^,^5^,^28^. Towards this end, it is important to understand the lineage derivation or derivations of newly proliferated astrocytes in CNS lesions.

In the present study, we tested the generality of the proposal that ependymal cells represent a major source of adult neural stem cells that provide the majority of newly proliferated scar-forming astrocytes that protect tissue and function after SCI^22^,^23^,^24^,^25^. We quantified the distribution and molecular phenotype of ependymal cell progeny in SCI lesions generated by different SCI models, including severe full crush injuries encompassing the entire spinal cord, as well as small precise stab injuries that did or did not directly damage the ependyma. We studied young adult mice using a knock-in *Foxj1*^*CreERT2:GFP*^ reporter based fate mapping strategy^29^, combined with BrdU labeling of newly proliferated cells, immunofluorescence of cell-type specific molecular markers and quantitative morphometric analyses. In contrast with the previous reports^22^,^23^,^24^,^25^, we found no evidence that ependymal cells are a major source of endogenous adult neural stem cells or generate substantial numbers of molecularly verified astrocytes after SCI.

## Results

### Foxj1^CreERT2^ targeting of reporter protein to uninjured ependyma

**Figure 1:**
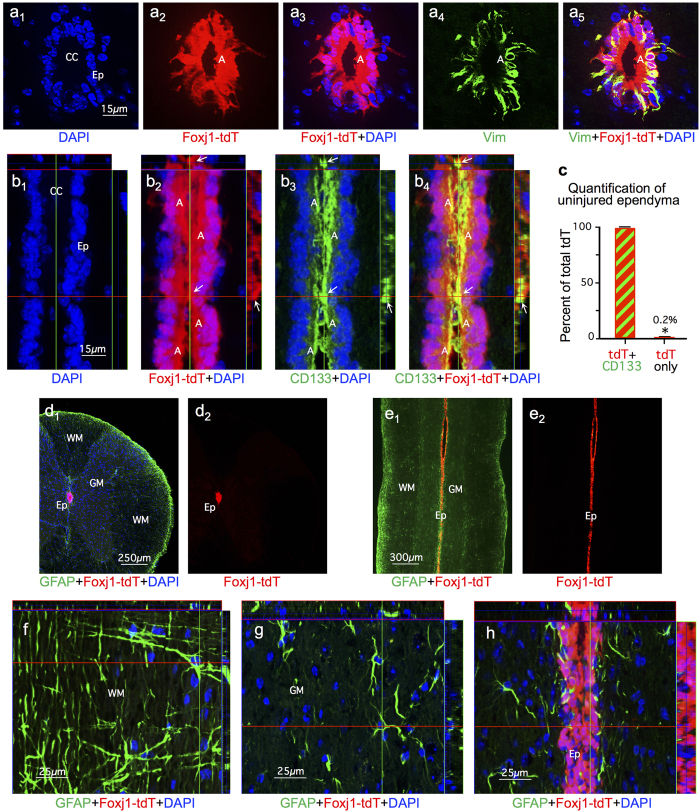
*Foxj1*^*CreERT2*^-tdT (Foxj1-tdT) expression is confined to molecularly confirmed ependymal cells in uninjured adult murine spinal cord. (a_1–5_, b_1–4_) Single channel and merged immunofluorescence images of transverse (**a**) or horizontal (**b**) sections through uninjured spinal cord ependyma (Ep) and central canal (CC). (a_1_-a_5_) Note that all ependymal cells with apical membranes (A) in contact with the CC lumen express Foxj1-tdT in those apical membranes and adjacent cytoplasm (A) and these Foxj1-tdT cells also express vimentin (Vim) in their central and basal cell portions and in some radial processes. (b_1_-b_4_) Note that all ependymal cell apical membranes (A) in contact with CC lumen are intensely co-labeled with both Foxj1-tdT and CD133 (arrows), which is also present but less intense in adjacent apical cytoplasm (A). (**c**) Graph comparing the percent of overlap of Foxj1-tdT and CD133 in the ependymal cell layer. n = 4 per group, *p < 0.001 (t-test). (**d**–**g**) Transverse and horizontal images showing Foxj1-tdT labeled ependyma and GFAP-immunoreactive astrocytes uninjured spinal cord. Note the complete absence of Foxj1-tdT labeled cells from all regions of grey (GM) and white matter (WM) and that no astrocytes express Foxj1-tdT.

To target CNS ependymal cells for fate mapping of progeny generated after SCI, we used mice with CreERT2 inserted into the Foxj1 locus^29^ crossbred with tdTomato (tdT) reporter mice^30^. To characterize this *Foxj1*^*CreERT2*^-tdT lineage analysis model, denoted henceforth as Foxj1-tdT, we determined which cells exhibited tdT reporter expression after tamoxifen induction in uninjured mice. In the absence of tamoxifen, there was no detectable tdT expression (not shown). In uninjured adult mice given tamoxifen and evaluated after drug washout, tdT was clearly expressed by essentially all ependymal cells defined as ciliated cells with apical surfaces contacting the central canal lumen^22^,^31^,^32^ (Fig. 1a–c). The ependymal marker CD133, which labels ciliated cells^31^,^32^, was expressed by essentially all Foxj1-tdT expressing ependyma (Fig. 1b,c). Notably, Foxj1-tdT and CD133 were intensely co-localized to all ependymal cell apical membranes in direct contact with the central canal lumen (Fig. 1b); CD133 was also detectable (though less intensely so) in the immediately adjacent apical cytoplasm (Fig. 1b). Vimentin, another ependymal cell marker^31^,^32^, was detectably expressed by nearly all Foxj1-tdT expressing cells, but in contrast with CD133 was absent from apical cell portions and was instead present in central and basal cell portions and radial processes (Fig. 1a). CD133 was expressed by a number perivascular cells, whereas vimentin was not detectable in other cell types in uninjured spinal cord as described previously^31^,^32^. No tdT expression could be detected outside the ependymal cell layer (Fig. 1d,e), and there was no detectable tdT expression in GFAP-positive astrocytes or any other cell types in spinal cord grey or white matter (Fig. 1d–h). These findings demonstrated this Foxj1-tdT model labeled essentially all ependymal cells and no other spinal cord cell types, and is thus appropriate for fate mapping the progeny of ependymal cells derived after SCI in adult mice.

### Fate mapping of ependymal progeny after full transverse crush SCI

**Figure 2:**
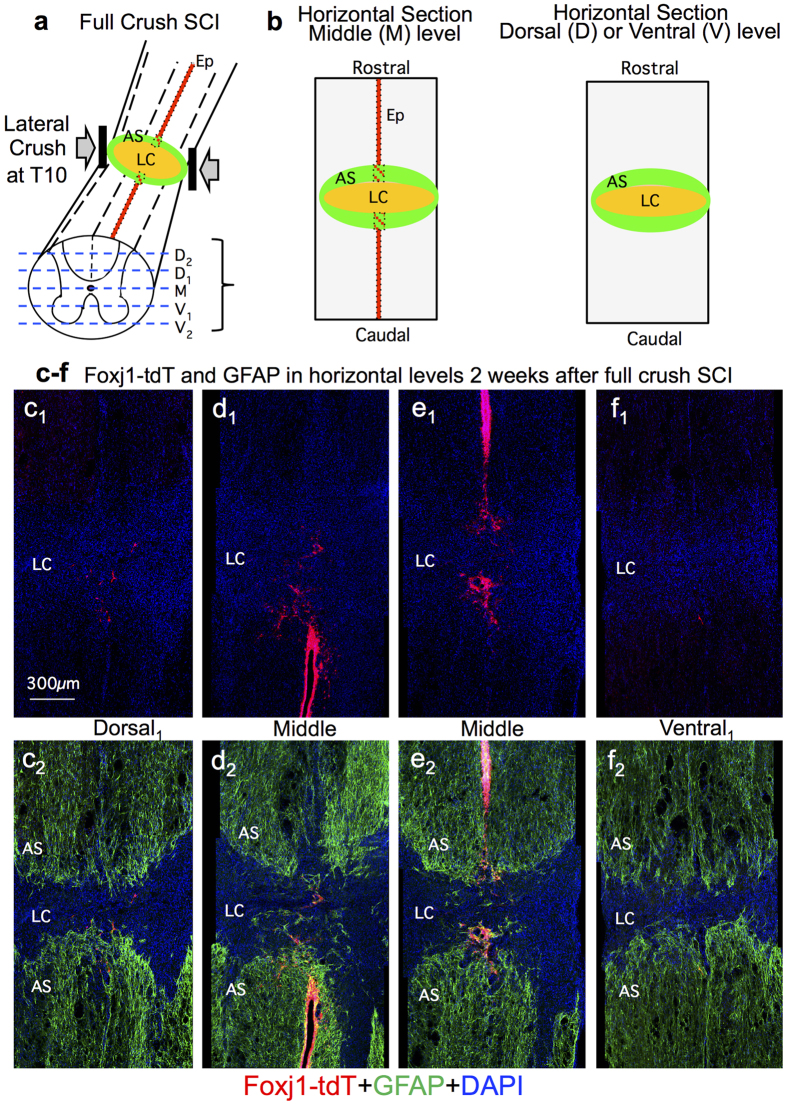
At 2 weeks after a full transverse crush SCI, Foxj1-tdT expressing cells have not migrated into lesions and remain primarily near the ependymal layer. (**a**) Schematic of crush SCI and 5 dorso-ventral levels of horizontal sections used for qualitative and quantitative analyses. (**b**,**c**) Schematics of horizontal views of middle level (M) and dorsal (D) or ventral (V) levels showing ependyma (Ep) as well as demarcations of astrocyte scar (AS) and lesion core (LC) used for analyses. (**c**–**f**) Single channel and merged immunofluorescence images showing distribution of Foxj1-tdT positive ependymal cells and their progeny in relation to the LC and to GFAP positive astrocytes in the AS.

We next examined the contribution of Foxj1-tdT ependymal cell progeny to the proliferative wound response after severe transverse crush SCI across the entire spinal cord. Adult uninjured *Foxj1*^*CreERT2*^-tdT mice were induced with tamoxifen and given a full transverse crush SCI at T10 (Fig. 2a). BrdU was administered to label mitosis induced by the SCI. Tissue was collected after 2 and 8 weeks and was quantitatively evaluated in horizontal tissue sections at 5 dorso-ventral levels (Fig. 2a–c). These time points were chosen because by 2 weeks after SCI, astrocyte scars are fully formed by newly proliferated astrocytes and by 8 weeks after SCI these astrocyte scars are fully mature and somewhat more compact^7^,^20^. The well-established peak period of astrocyte proliferation occurs during the first week after SCI in rodents, and thereafter few new astrocytes are generated^7^,^33^,^34^.

**Figure 3:**
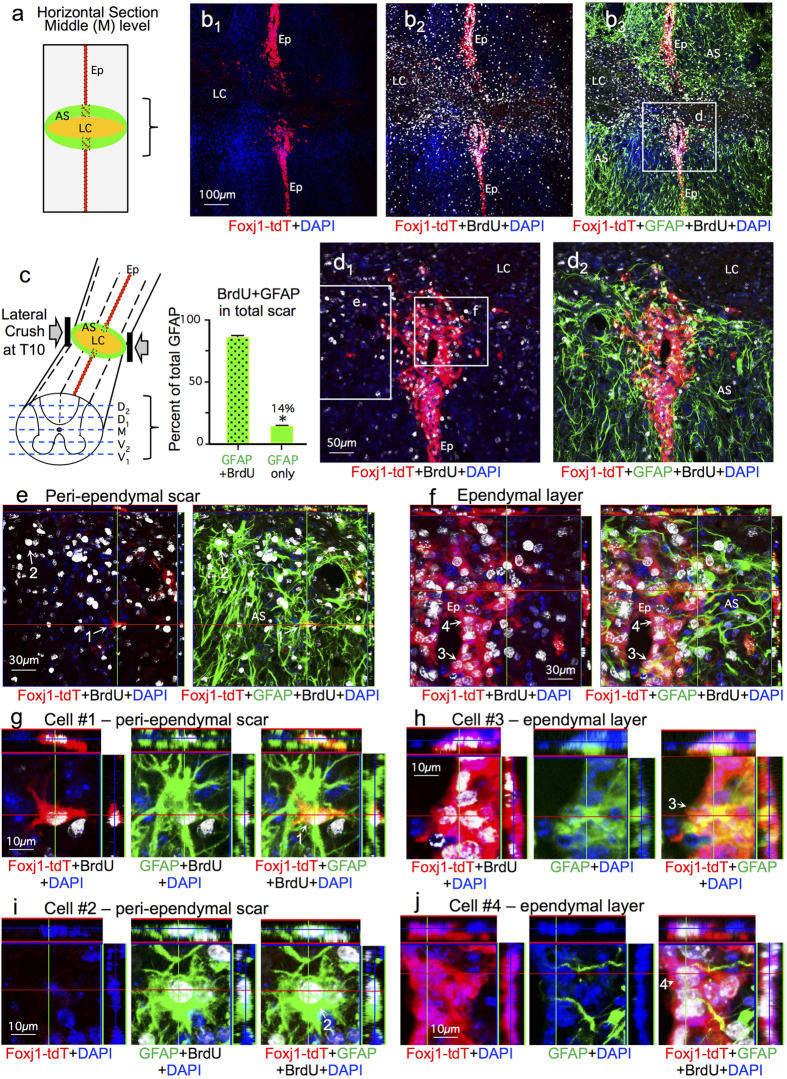
At 2 weeks after crush SCI, there is substantial BrdU labelling of many cells, including a majority of GFAP positive scar-forming astrocytes and many Foxj1-tdT positive ependyma. (**a**,**b**) Schematic of SCI lesion area shown and survey images of single channel and merged immunofluorescence comparing cells labeled for BrdU, Foxj1-tdT and GFAP in the lesion core (LC), ependyma (Ep) and astrocyte scar (AS). (**c**) Schematic of 5 dorso-ventral levels quantified and graph showing the percent of GFAP positive scar-forming astrocytes that are BrdU labeled and newly proliferated 2 weeks after SCI. n = 6 per group, *p < 0.001 (t-test). (**d**) Higher magnification of box in b showing the ependymal region and astrocyte scar adjacent to the lesion core. (**e**,**f**) Higher magnification orthogonal images of boxes in d showing many BrdU labeled cells positive for GFAP or Foxj1-tdT. (**g**–**j**) Detail orthogonal images of cells #1–4 labeled in (**e**,**f**) comparing the overlap of staining for GFAP and Foxj1-tdT within the ependymal layer and adjacent astrocyte scar.

At 2 weeks after full crush SCI, tissue lesions spanned the entire transverse spinal cord at all dorso-ventral levels and exhibited the expected appearance of a central lesion core of non-neural tissue surrounded by scar forming astrocytes with extensively overlapping processes (Fig. 2c–f)^7^,^20^. Qualitative analysis at multiple dorso-ventral levels indicated that Foxj1-tdT labeled cells were concentrated within the ependymal layer. A small number of scattered tdT labelled cells were also present in the immediate vicinity of the ependyma damaged by SCI lesion, but only very few tdT labelled cells had migrated into other portions of the SCI lesion (Figs 2c–f and 3a,b). For quantitative analyses, we examined separately either the primarily non-neural lesion core, or in the immediately surrounding 500 μm astrocyte scar border zones (Fig. 2b,c)^7^. Over 85% of the GFAP positive cells in these scar borders were labeled with the current regimen of twice daily BrdU pulses labeled (Fig. 3c), confirming that the overwhelming majority of scar forming astrocytes are newly proliferated after SCI.

**Figure 4:**
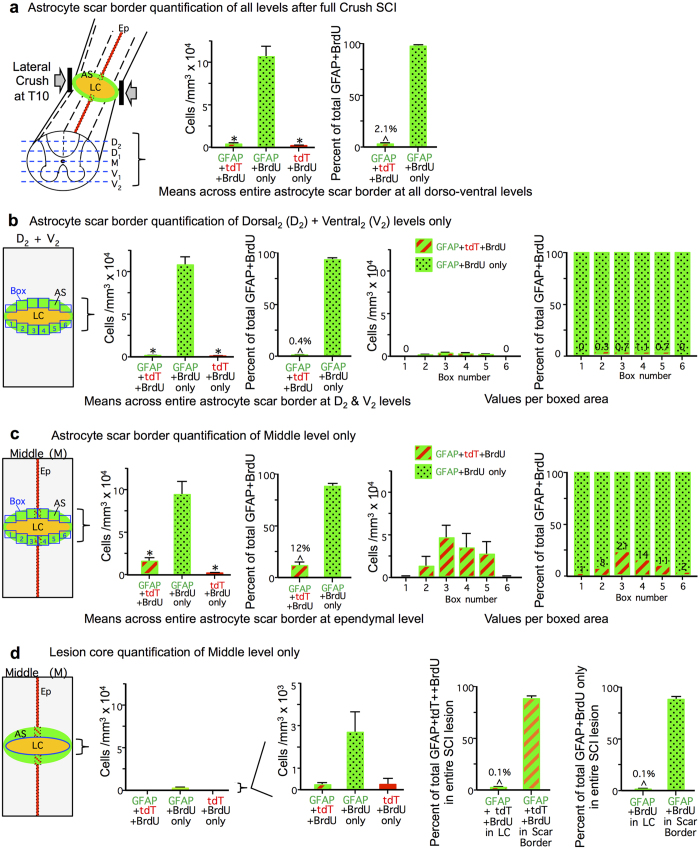
Quantification of BrdU positive cells co-labeled with either GFAP or Foxj1-tdT, or both in different regions of the lesion at 2 weeks after SCI. (**a**) Schematic of 5 dorso-ventral levels quantified and graphs showing the total number of cells per tissue volume and mean percent of BrdU labeled cells that are co-labeled with GFAP or Foxj1-tdT or both across the entire astrocyte scar (AS). Total values were derived by averaging counts from 6 boxes across the entire transverse spinal cord at each of the 5 levels as shown in (**b**,**c**). (**b**) Schematic of 6 counting boxes evaluated across the transverse spinal cord at dorsal and ventral levels D_2_ and V_2_. Graphs show the mean number of cells per volume and mean percent of BrdU labeled cells that are co-labeled with GFAP or Foxj1-tdT or both across the entire transverse plane, as well number or percent of cells per box. (**c**) Schematic of 6 counting boxes evaluated across the transverse spinal cord at the middle (M) level of the ependymal layer (Ep). Graphs show the mean number of cells per volume and mean percent of BrdU labeled cells that are co-labeled with GFAP or Foxj1-tdT or both across the entire transverse plane, as well number or percent of cells per box. (**d**) Schematic of lesion core (LC) at the middle (M) level containing the ependymal layer (Ep). Graphs show the total number per volume of BrdU labeled cells that are co-labeled with GFAP or Foxj1-tdT or both across the entire transverse plane, as well the percent of such cells in the entire SCI lesion that are located in either the lesion core or astrocyte scar. n = 6 per group, *p < 0.001 versus GFAP + BrdU only (ANOVA with Newman-Keuls), ^p < 0.001 (t-test).

We then counted BrdU labeled cells that were labeled with either tdT plus GFAP (Fig. 3g,h), GFAP alone (Fig. 3i) or tdT alone (Fig. 3j). Only 2.1% of all BrdU labeled and GFAP-positive cells were tdT positive in scar borders across the entire SCI lesion within these 5 dorso-ventral levels (Fig. 4a). This minimal contribution of ependymal progeny to newly proliferated cells generated after SCI was surprising to us in light of the previous reports that large numbers of virally and transgenically fate mapped ependymal cell progeny were generated that migrated extensively into SCI lesions and contributed the majority of newly generated astrocytes in such lesions^22^,^23^,^24^,^25^. We therefore investigated various factors that might underlie the striking difference between our results and these previous reports.

We began by examining the distribution of Foxj-1-tdT fate mapped ependymal cell progeny in different portions of the SCI lesion. Qualitative evaluation suggested that the majority of tdT positive ependymal cell progeny after SCI were in close proximity to the damaged ependymal cell layer and appeared to have sealed off the damaged central canal (Fig. 3d,f) and that very few of such cells had migrated appreciable distances into other portions of the SCI lesion (Figs 2c–f and 3b,d). To test this observation quantitatively, we counted cells that were BrdU positive and also labeled with GFAP, tdT, or both in a series of equally sized boxes that spanned across the entire spinal cord and covered the 500 μm scar border zone immediately surrounding severe crush SCI lesions^7^. We found a decreasing gradient of cells positive for both Foxj1-tdT and GFAP as distance from ependyma increased in both the medio-lateral and dorso-ventral planes. Boxes furthest from ependyma in both planes contained no such cells at all (Fig. 4b). Even within the two central quantification boxes that directly contained the damaged ependymal layer, the relative number of cells positive for both Foxj1-tdT and GFAP ranged only from 14 to 21% (Fig. 4c). Notably, many of these cells triple labeled for BrdU, Foxj1-tdT and GFAP were clearly part of the ependymal layer in direct contact with the central canal (Fig. 3h) and had morphologies indistinguishable from those of adjacent GFAP-negative, Foxj1-tdT positive ependymal cells (Fig. 3j). In this regard it is noteworthy that as reported previously, GFAP is expressed in small numbers of uninjured ependymal cells^32^ (Fig. 1h).

We also examined Foxj1-tdT cells in the lesion core. Both qualitative and quantitative analyses indicated minimal representation of Foxj1-tdT positive ependymal progeny in SCI lesions cores, whether they were GFAP-positive or not, and such cells were present only in the immediate vicinity of damaged ependyma (Figs 2c–f, 3b,d and 4d).

We next examined the spinal cord 8 weeks after severe crush SCI. Previous reports suggested that the relative proportion of scar-forming astrocytes derived from ependymal progenitors increased over time until they comprised the major source of such cells^23^. This assertion seemed at odds with well-established observations from multiple laboratories that the majority of newly proliferated astrocytes are generated during the first week after rodent SCI or brain injury, and that only minimal new astroglia are added to injuries over subsequent weeks^7^,^33^,^34^,^35^. To re-examine this concept, we administered BrdU continually after SCI until tissue was harvested.

**Figure 5:**
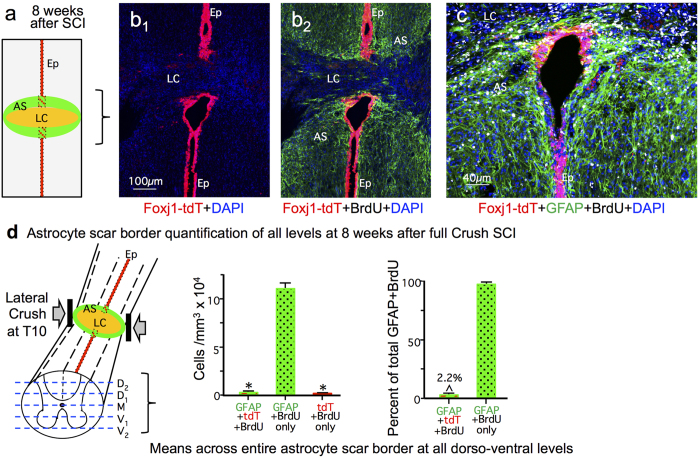
At 8 weeks after crush SCI, labelling for BrdU, Foxj1-tdT and GFAP remain qualitatively and quantitatively similar to that seen at 2 weeks after SCI. (**a**) Schematic of SCI lesion area shown in (**b**,**c**). (**b**) Single channel and merged immunofluorescence images showing distribution of Foxj1-tdT positive cells and their progeny in relation to the ependymal layer (Ep), lesion core (LC) and GFAP positive astrocytes in the astrocyte scar (AS). (**c**) Higher magnification view of cells labeled for BrdU, Foxj1-tdT and GFAP in the lesion core, ependyma and astrocyte scar. (**d**) Schematic of 5 dorso-ventral levels quantified and graphs showing the total number of cells per tissue volume and mean percent of BrdU labeled cells that are co-labeled with GFAP or Foxj1-tdT or both across the entire astrocyte scar, determined from cells counts conducted in an identical manner as at 2 weeks after SCI. n = 3 per group, *p < 0.001 versus GFAP + BrdU only (ANOVA with Newman-Keuls), ^p < 0.001 (t-test).

Qualitative examination of BrdU-positive and Foxj1-tdT-labeled cells at 8 weeks after SCI (Fig. 5a–c) was indistinguishable from that at 2 weeks (Figs 2c–f and 3b,d). Specifically, at 8 weeks after SCI, as at 2 weeks, the majority of tdT positive ependymal cell progeny were found in close proximity to the damaged ependymal cell layer, where they appeared to have sealed off the damaged central canal, and very few ependymal cells had migrated appreciable distances into other portions of the SCI lesion (Fig. 5a–c). Quantification showed similar numbers of BrdU labeled cells labeled with GFAP, tdT, or both in the total scar border at 8 weeks (Fig. 5a–d), and at 2 weeks (Fig. 4a) after SCI, such that at both time points ependymal cell progeny positive for BrdU, tdT and GFAP represented only about 2% of total population of cells positive for BrdU and GFAP (Figs 4a and 5d). It deserves emphasis that this percent value cannot be automatically equated with the number of newly generated astrocytes that might have derived from putative ependymal progenitors for two reasons. First, many Foxj1-tdT and GFAP cells were part of the ependymal layer and indistinguishable for ependymal cells labeled only for Foxj1-td and CD133 (Fig. 3h,j), rendering them unlikely to be astrocytes, and more likely to be ependyma^32^. Second, because our sampling procedure began in the spinal cord center through the narrow ependymal layer and Foxj1-tdT cells did not migrate far from this region, our counts in 5 evenly spaced dorso-ventral levels would over represent such cells relative to 5 randomly selected but evenly spaced sections that might not always include the center of the ependyma. Notably at 8 weeks after SCI, the central canal, which was discontinuous across the severe lesion, had been sealed by new ependyma on both sides of the lesion (Fig. 5b,c). Thus, our findings strongly contradicted previous reports that putative ependymal progenitors generate the majority of scar forming astrocytes in SCI lesions, and instead suggest that ependymal cells may proliferate in particular to repair and seal off the damaged central canal.

### Fate mapping of ependymal progeny after lateral or midline stab injury

**Figure 6:**
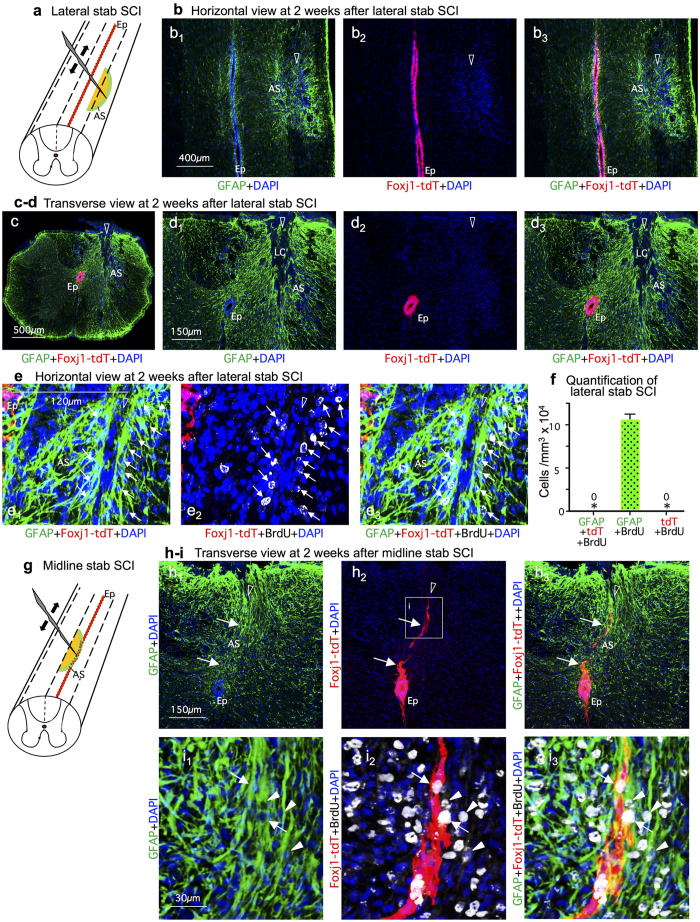
At 2 weeks after dorsal stab SCI, Foxj1-tdT expressing cells migrate into lesions only when the ependymal layer is directly injured. (**a**) Schematic of lateral stab SCI not damaging ependyma (Ep). (**b**) Single channel and merged immunofluorescence images of Foxj1 and GFAP in a horizontal section through a lateral stab SCI that is at level of the ependymal layer but is lateral to and does not damage ependyma. No Foxj1-tdT cells are present in the astrocyte scar (AS). (**c**,**d**) Single channel and merged immunofluorescence images of of Foxj1 and GFAP in a transverse section through a lateral stab SCI that penetrates to the level of the ependymal layer but remains lateral to and does not damage ependyma. No Foxj1-tdT cells are present in the astrocyte scar or lesion core (LC). (**e**) Single channel and merged immunofluorescence images of Foxj1, GFAP and BrdU in a horizontal section through a lateral stab SCI that is only 120 μm away from, but does not damage ependyma, and contains no Foxj1-tdT cells in the astrocyte scar (arrows). (**f**) Mean number of cells per volume of BrdU labeled cells that are co-labeled with GFAP or Foxj1-tdT or both in lateral stab injuries that do not damage ependyma. n = 6 per group, *p < 0.001 versus GFAP + BrdU only (ANOVA with Newman-Keuls). (**g**) Schematic of medial stab SCI directly damaging the ependyma. (**h**) Single channel and merged immunofluorescence images of of Foxj1 and GFAP in a transverse section through a medial stab SCI that penetrates into and directly damages ependyma, resulting in Foxj1 cells along the astrocyte scar (arrows). (**i**) Higher magnification of boxed area in h showing newly proliferated cells labelled for BrdU, Foxj1 and GFAP (arrows) or for BrdU and GFAP only (arrowheads) along the astrocyte scar.

To determine what might underlie the large differences of our findings with previous reports with respect to ependymal cell contribution to astrocyte production after SCI^22^,^23^,^24^, we first compared SCI models. The previous studies were all based on radially oriented midline stab injuries, which in the images shown penetrate to the level of the ependyma^22^,^23^,^24^. These same previous studies reported that in uninjured spinal cord, ependyma give rise to few if any cells that migrate into normal parenchyma to replace neural lineage cells^22^,^23^. These observations together with our findings after crush injury suggested that ependyma might generate progeny cells only after direct ependymal injury. To test this possibility, we placed stab injuries into the lateral spinal cord that penetrated to the depth of the ependyma but remained lateral to the ependyma and did not contact or damage the ependyma (Fig. 6a–e). Such injuries contained large numbers of newly proliferated, scar-forming astrocytes that were positive for both BrdU and GFAP, but never contained any tdT positive cells, with or without BrdU, (Fig. 6a–f), even when the lesions came to within less than 150 μm of the lateral edge of the ependymal layer (Fig. 6e). For comparison, a different group of Foxj1-tdT mice received radially oriented stab SCI along the spinal cord midline that penetrated to the ependyma. In agreement with the previous studies^22^,^23^,^24^, these mice exhibited tdT positive cells extending up from the ependymal layer into and along the margins of the directly contiguous stab injury (Fig. 6g–i). Many of these cells were positive for BrdU as well as tdT and many were also positive for GFAP (Fig. 6g–i). Taken together, these findings clearly demonstrated that ependymal cells do not contribute any cells to the CNS wound response unless the ependyma themselves are directly injured.

### Comparison of molecular markers to identify astrocytes after SCI

We next asked whether the large differences of our findings with previous reports might also be due to differences in the molecular markers used to identify astrocytes. We noted that previous studies based their conclusions that ependymal cells give rise to the majority of scar-forming astrocytes in SCI lesions on the assertion that the majority of those putative astrocytes were “Sox9 positive, vimentin positive and GFAP negative”^22^,^23^,^24^. However, both Sox9^36^,^37^ and vimentin^32^,^38^,^39^ are highly expressed by ependymal cells, raising questions as to the validity of using these markers in the absence of GFAP to identify ependymal progeny as astrocytes. In addition, there is no precedent in the literature for the existence of scar-forming reactive astrocytes that do not express GFAP. We therefore examined more closely the molecular phenotype of Foxj1-tdT expressing cells and their progeny after SCI by comparing various combinations of widely accepted markers used to identify either astroglia or ependyma.

**Figure 7:**
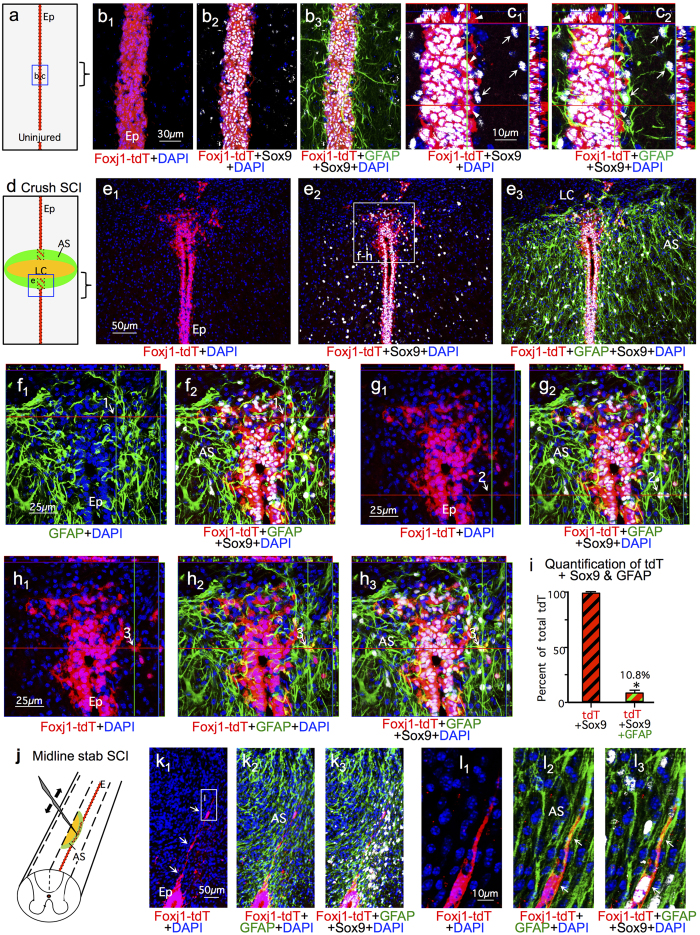
Sox9 is expressed by astrocytes that are GFAP positive and by ependyma that are Foxj1-tdT positive but GFAP negative. (**a**) Schematic of uninjured ependyma (Ep) with boxed region shown in (**b**,**c**). (**b**,**c**) Single channel and merged immunofluorescence images of Sox9, Foxj1 and GFAP in a horizontal section of uninjured ependymal layer. Sox 9 is present in either GFAP positive astrocytes (arrows) or Foxj1 positive ependyma (arrowheads) but no cells are positive for both GFAP and Foxj1. (**d**) Schematic of SCI crush lesion with box of peri-ependymal region shown in (**e–h**). (**e**) Single channel and merged immunofluorescence images showing Sox9, Foxj1 and GFAP in a horizontal section through the ependymal layer, lesion core (LC) and astrocyte scar (AS). (**f**–**h**) Higher magnification of boxed area in e showing cells labelled for Sox9, Foxj1 or GFAP in the ependymal layer and adjacent astrocyte scar. Numbers indicate cells labeled for (1) Sox9 and Foxj1-tdT only, (2) Sox9 and GFAP only, and (3) Sox9, Foxj1 and GFAP. (**i**) Mean percent of Sox9 labeled cells that are co-labeled with Foxj1-tdT alone or with both Foxj1-tdT plus GFAP across the entire SCI lesion. n = 4 per group, p < 0.001 (t-test). (**j**) Schematic of medial stab SCI directly damaging the ependyma as shown in (**k,l**). (**k**) Single channel and merged immunofluorescence images showing Sox9, Foxj1 and GFAP in a transverse section through a medial stab SCI that penetrates into and directly damages ependyma, resulting in Foxj1 cells along the astrocyte scar (arrows). (**l**) Higher magnification of boxed area in k showing cells labelled for Sox9, Foxj1 and GFAP (arrows) in the astrocyte scar.

**Figure 8:**
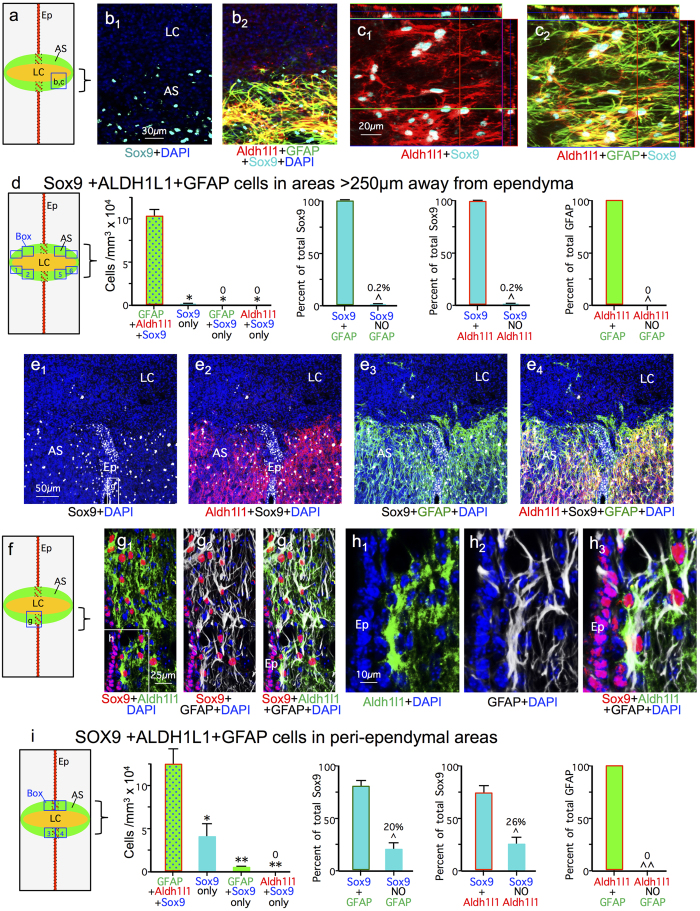
No Sox9 positive cells express the astrocyte marker Aldh1l1 in the absence of GFAP. (**a**) Schematic of SCI crush lesion with boxed region of lateral portion of astrocyte scar (AS) shown in **b,c**. (**b**,**c**) Single channel and merged immunofluorescence images showing complete overlap of staining for Sox9, GFAP and Aldh1l1 in a horizontal section through the lateral astrocyte scar. (**d**) Schematic of SCI crush lesion showing the 4 counting boxes evaluated in the lateral portions of the astrocyte scar. Graphs show the mean number of cells per volume and mean percent of total Sox9 or total GFAP labeled cells that are co-labeled with Sox9, GFAP or Aldh1l1 in the lateral astrocyte scar. n = 6 per group, *p < 0.001 versus GFAP + Aldh1l1 + Sox9 (ANOVA with Newman-Keuls), ^p < 0.001 (t-test). (**e–g**) Single channel and merged immunofluorescence images showing Sox9, GFAP and Aldh1l1 in a horizontal section through the ependymal layer (Ep), lesion core (LC) and astrocyte scar. (**f**) Schematic of SCI crush lesion with boxed region of ependyma and astrocyte scar shown in (**g,h**). (**g**) Higher magnification of boxed area in **e**, showing ependymal cells positive for Sox9 only and astrocytes positive for Sox9, GFAP and Aldh1l1. (**h**) Higher magnification of boxed area in g. Note in (**e-g**) that all cells positive for Sox9 and Aldh1l1 are also positive for GFAP. (**i**) Schematic of SCI crush lesion showing the 4 counting boxes evaluated in the medial portions of the astrocyte scar adjacent to and including the ependyma. Graphs show the mean number of cells per volume and mean percent of total Sox9 or total GFAP labeled cells that are co-labeled with Sox9, GFAP or Aldh1l1 in the lateral astrocyte scar. n = 6 per group, *p < 0.01, **p < 0.001 versus GFAP + Aldh1l1 + Sox9 (ANOVA with Newman-Keuls), ^p < 0.01, ^^p < 0.001 (t-test).

**Figure 9:**
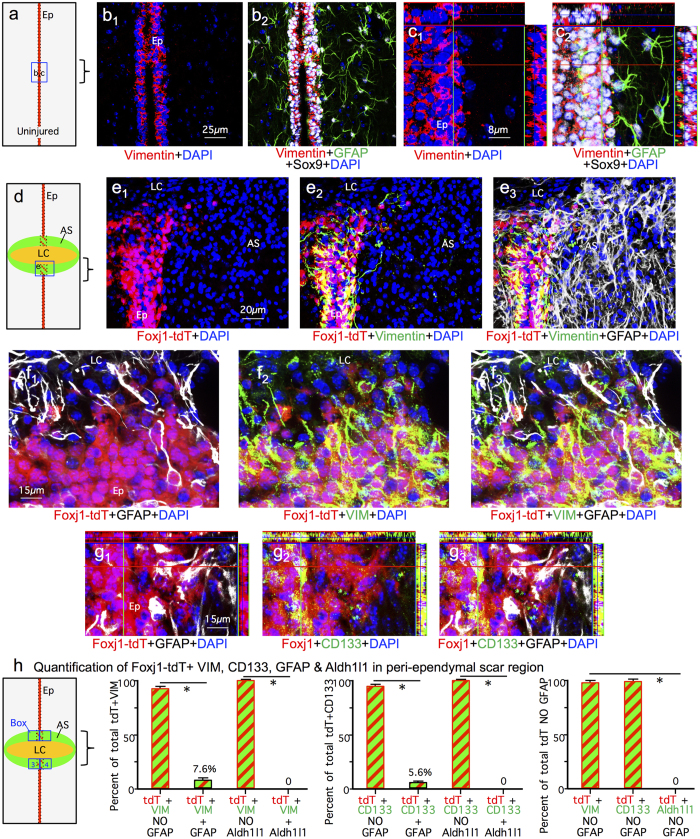
Comparison of Sox9, vimentin, CD133, Foxj1-TdT, GFAP and Aldh1l1 in the ependymal layer and adjacent regions in uninjured spinal cord and after crush SCI. (**a**) Schematic of uninjured ependyma (Ep) with boxed region shown in (**b**,**c**). (**b**,**c**) Single channel and merged immunofluorescence images of Sox9, vimentin and GFAP in a horizontal section of uninjured ependymal layer. Sox 9 is present in both GFAP positive astrocytes (arrows) and vimentin positive ependyma (arrowheads) but no cells are positive for both GFAP and vimentin. (**d**) Schematic of SCI crush lesion with box of peri-ependymal region shown in (**e**–**g**). (**e**,**f**) Single channel and merged immunofluorescence images showing Foxj1, vimentin and GFAP staining in horizontal sections through the ependymal layer, lesion core (LC) and astrocyte scar (AS). (**e**) Vimentin co-localizes with Foxj1-tdT ependyma but is low or undetectable in GFAP positive scar forming astrocytes adjacent to the ependymal layer. (**f**) Most Foxj1-tdT cells in the ependymal layer adjacent to the lesion core express vimentin, and a few express GFAP. (**g**) Single channel and merged immunofluorescence images showing Foxj1, CD133 and GFAP staining in horizontal sections through the ependymal layer adjacent to lesion core. Most Foxj1-tdT cells in the ependymal layer adjacent to the lesion core express CD133, and a few express GFAP. (**i**) Schematic of SCI crush lesion showing the 4 counting boxes evaluated in the medial portions of the astrocyte scar adjacent to and including the ependyma. Graphs show the mean percent of total Foxj1-tdT + vimentin cells or total Foxj1-tdT + CD133 that are also co-labeled with GFAP or Aldh1l1; or the mean percent of the total number of Foxj1-tdT positive and simultaneously GFAP negative cells that are co-labeled with either vimentin, CD133 or Aldh1l1. n = 3 per group, *p < 0.001 (ANOVA with Newman-Keuls).

To identify ependyma we used CD133^31^,^32^ and vimentin^38^,^39^, while reactive astrocytes were identified by both GFAP and Aldh1l1 labeling. GFAP was first isolated from CNS lesions^40^ and has over many decades of research become the canonical maker of astrocyte reactivity in response to CNS damage. There is a long history of evidence that GFAP is expressed by essentially all reactive astrocytes^6^,^41^,^42^. Nevertheless, we additionally probed the molecular phenotype of Foxj1-tdT ependymal cell progeny that might be putative astrocytes after SCI by staining for Aldh1l1, which is widely regarded as a reliable marker for most if not all astrocytes including non-reactive astrocytes that express low or undetectable levels of GFAP in healthy CNS tissue^43^,^44^,^45^. Although vimentin can be expressed by some scar forming astrocytes, the expression level and staining intensity is far lower than that of nearby ependymal cells^13^. Accordingly, we examined the overlap of Foxj1-tdT labeled cells with staining for either Sox9, GFAP, Aldh1l1, vimentin or CD133 in uninjured mice and after crush or stab SCI (Figs 7, 8 and 9).

In uninjured animals, essentially all Foxj1-tdT labeled ependymal cells robustly expressed Sox9 (Fig. 7a–c), vimentin (Fig. 9a–c) and CD133 (Figs 1f,g and 9g,h). In addition, uninjured tissue immediately adjacent to ependyma contained many GFAP expressing astrocytes that expressed Sox9 but not Foxj1-tdT (Fig. 7b,c) or vimentin (Fig. 9b,c) or CD133 (not shown).

After crush SCI, essentially all Foxj1-tdT positive cells within the ependymal layer continued to express both Sox 9 (Fig. 7d–i), vimentin (Fig. 9d–f,h) and CD133 (Fig. 9g,h). It is also noteworthy that lesion core areas after crush SCI contained many newly proliferated BrdU labeled cells (Fig. 3b), but contained few cells positive for Foxj1-tdT (Figs 2c–f and 3b) or Sox9 (Fig. 8e).

Since previous reports suggested that fate-mapped ependymal cells gave rise to substantial numbers of putative astrocytes that were Sox9 positive but GFAP negative after SCI^22^,^23^,^24^, we characterized in detail the molecular phenotypes Sox9 and Foxj1-tdT expressing cells in different regions of crush SCI lesions. In the lateral scar border after crush SCI, which contained few or no Foxj1-tdT positive cells (Figs 2c and 3b), 99.8% of Sox9 positive cells were also positive for both GFAP and Aldh1L1 (Fig. 8a–d). In contrast, in medial regions of scar that included the damaged ependymal layer and its immediately vicinity, about 20% of Sox9 positive cells were negative for GFAP (Fig. 8e–i). The majority of these Sox9 positive but GFAP negative cells were directly within the ependymal layer or in its immediate vicinity (Fig. 8e–h), and most of these cells were positive for vimentin or CD133 (Fig. 9f–h). Most of these cells had no obvious contact with the central canal lumen and exhibited no detectable apical-basal polarity, but instead expressed vimentin or CD133 staining throughout their cytoplasm (Fig. 9f–h). In addition, it is important to note that all Aldh1l1 positive cells in peri-ependymal scar areas were also positive for GFAP and Sox9 (Fig. 8e–h) or GFAP and Foxj1-tdT (Fig. 9h). Thus, we found no evidence for the existence of cells that were positive for either Sox9 or Foxj1-tdT and that were simultaneously negative for GFAP but positive for Aldh1l1.

After midline stab SCI, scar regions in direct contact with the damaged ependymal layer contained small numbers of cells positive for Foxj1-CreERT-tdT, Sox9 and GFAP (Fig. 7j–l). In contrast with previous reports, we found no evidence for large numbers of cells that were positive for Foxj1-tdT or Sox9 and simultaneously negative for GFAP other than cells directly within the ependymal layer (Fig. 7k,l).

These findings demonstrate that after SCI, there are no cells that are positive for Sox9 or Foxj1-tdT and simultaneously negative for GFAP that meet well established molecular criteria for being astrocytes. Instead we found that such cells express markers associated with ependyma or potentially some non-astrocyte ependyma progeny.

## Discussion

In this study we show that, (i) after severe SCI involving the entire spinal cord, ependymal cell progeny contribute less than 2%, and more probably none, of the total newly proliferated molecularly validated astrocytes by staining for GFAP and Aldh1l1, (ii) ependymal cell contribution to other cell types after SCI is also minimal, (iii) ependymal progeny fail to migrate substantially into SCI lesions and remain primarily in the immediate vicinity of the damaged ependymal layer around the central canal, and (iv) ependymal progeny do not contribute any cells to nearby SCI lesions that do not directly damage ependyma. Thus, our findings do not support previous reports that after SCI, ependyma are a major source of endogenous neural stem cells that migrate extensively to and throughout lesions and provide the majority of neuroprotective astrocytes^22^,^23^,^24^,^25^. The contradictions between our and these previous findings are most likely due to fundamental differences in experimental injury models and the types of molecular markers used to claim identification of specific cell phenotypes, and potentially to differences in cell fate mapping strategies.

Previous studies reporting ependyma as major neural stem cells after SCI used two transgenically-targeted fate mapping strategies with an unusual Nestin-CreERT line and a human FOXJ1-CreERT line^22^,^23^,^24^,^25^. The Nestin-CreERT line these studies used exhibits an unexplainable recombination seemingly restricted to ependymal cells that appears fundamentally different from the far more widespread recombination patterns of other Nestin-CreERT lines^46^,^47^. Conversely, the same human promoter in the FOXJ1-CreERT line used previously^22^,^23^,^24^,^25^ drives forebrain recombination patterns in FOXJ1-GFP mice^48^ that are more widespread than those seen with knock-in of CreERT2 into the mouse *Foxj1* locus^29^. Thus, it cannot be ruled out that the unusual recombination patterns observed in the human FOXJ1-CreERT line impacted on reporter gene expression after SCI. To avoid such possible confounds, we used the knock-in *Foxj1*^*CreERT2*^ line^29^ to drive reporter gene expression, thus ensuring that fate mapping model was conducted while remaining faithful to the activity of the endogenous Foxj1 locus, which in the murine CNS is largely confined to ependyma. We confirmed that pulse delivery and wash out of tamoxifen in uninjured adult mice of this line induced robust tdT reporter expression in all ependyma and essentially no other detectable cells in spinal cord, validating the use of this model to fate map progeny of adult ependymal cells in murine SCI models.

Choice of SCI models can influence interpretation and extrapolation of experimental observations. Small stab SCI can be useful to study blood-spinal cord-barrier damage or to assess immediately local cellular and molecular responses, but not all observations may be generalizable to larger SCI lesions generated by full transverse crush or contusion. The previous proposal that ependyma are a major source of endogenous neural stem cells after SCI was extrapolated from studies using a specialized SCI model of stab injuries that were placed longitudinally along the spinal cord midline and that penetrated radially into the ependymal layer, thereby directly injuring ependymal cells, as evident from the photomicrographs provided^22^,^23^,^24^,^25^. Laterally placed injuries used as controls, were also radial in their trajectories and clearly penetrated into the ependyma in the images provided. In contrast, we show here that after severe crush SCI that traverses and involves the entire spinal cord, ependymal cell progenitors contribute minimal numbers of newly proliferated astrocytes or other cells to the overall SCI wound response. Our findings are consistent with observations by others who used the same Nestin-CreERT line as in previous studies^22^,^23^ to label and trace ependymal cell progeny after a complete transverse crush SCI, but found little or no qualitative evidence for contribution of ependymal progeny to overall astrocyte scar formation^26^. Moreover, others using similar fate mapping strategies show that ependymal cells do not provide any progeny cells to newly proliferated astrocytes or other cell types in spinal cord autoimmune inflammatory lesions^49^. Our study extends these findings and provides detailed quantitative evidence that explains discrepancies across different SCI models. We show that when ependymal cells are directly damaged either by crush or stab injuries, they give rise to small numbers of fate-mapped, reporter expressing progeny. In large injuries that traverse the spinal cord, these cells remain in the immediate vicinity of the ependyma and do not migrate far into the lesions. In contrast, radially placed midline stab SCI that are geographically restricted can give the false impression that ependymal cell progeny contribute to the lesion scar, but their contribution is not substantive. When stab SCI are placed laterally so as to penetrate to the level of the ependyma but remain lateral to and not damage the ependyma, then there is no contribution of ependymal progeny to the lesion, even when the stab SCI comes to within less than 150 μm of the undamaged ependyma. Together these findings show that the previous suggestions that ependyma are major contributors of neural stem cells after SCI^22^,^23^,^24^,^25^, is an mistaken interpretation based on the sole use of stab SCI placed longitudinally along the spinal cord midline and penetrating into and directly injuring the ependymal layer along the entire SCI length with the consequent effect of exaggerating the perception of the contribution of ependymal progeny to the SCI wound response.

Molecular markers can be useful signatures to identify cell phenotypes. To identify specific cell types, we used multiple well-established markers to identify ependyma, Sox9, CD133, vimentin^31^,^32^,^36^,^38^,^39^ or astrocytes, GFAP, Aldh1l1^6^,^41^,^42^,^43^,^44^,^45^. Inexplicably, the previous proposals that the majority of neuroprotective reactive astrocytes derived from ependymal cell progeny after SCI were based on the contention that these putative astrocytes were “Sox9 positive, vimentin positive and GFAP negative”^22^,^23^,^24^,^25^. This contention is surprising given that it is well established that both Sox9^36^ and vimentin^38^,^39^ are highly expressed by ependymal cells and that there is no published precedent in the literature for the existence of reactive astrocytes that do not express GFAP. Here, in direct contrast with the previous proposal, we show that Foxj1-tdT and Sox9 positive but GFAP negative cells found after SCI are simply ependymal cells that continue to express two reliable ependymal markers, vimentin and CD133, and that none of these Foxj1-tdT and Sox9 positive but GFAP negative cells expressed Aldh1l1, a well-accepted astrocyte marker. Thus, after SCI there are no GFAP-negative, ependyma-derived reactive astrocytes, and the contribution of putative ependymal cell progenitors to newly proliferated and molecularly validated reactive astrocytes that express both GFAP and Aldh1l1 is minimal, if any. The observation of small numbers of newly proliferated Foxj1-tdT and GFAP-positive cells in the immediately vicinity of damaged ependyma is most likely due to the expression of GFAP by certain ependymal cells^32^.

The observations of our study have implications for others attempting to identify potential cellular manipulations to improve neural repair and functional outcome after SCI. Severe human SCI lesions have large cores of non-neural tissue and cysts^50^. Repopulating such lesion cores with neural cells derived either by cell grafting or by manipulation of endogenous progenitors is a potential therapeutic strategy that might support axon regrow or produce neurons that form relay circuits^2^,^3^,^4^,^5^. Understanding the derivation and regulation of neural lineage cells that take part in the proliferative wound response after SCI may help define their potential for endogenous manipulation or grafting to improve outcome. Ependymal cells have been proposed as endogenous adult neural stem cells that are a major source of newly proliferated neural lineage cells after SCI that migrate into lesions and give rise to the majority of neuroprotective reactive astrocytes^22^,^23^,^24^,^25^ and may thus represent good candidates for endogenous manipulation or as a source for cell transplantation. Our findings strongly contradict these claims, and instead are consistent with reports by others who find no evidence that ependyma serve as neural progenitors in spinal cord^26^ or forebrain^51^,^52^. Our findings provide detailed quantitative evidence that even though large SCI lesions contain many newly proliferated cells labeled with BrdU, negligibly few of these are positive for the reliable knock-in ependymal lineage marker, *Foxj1*^*CreERT2*^-tdT used here. We show that small numbers of ependymal progeny are generated only after direct damage, and these remain locally in the immediate peri-ependymal area. Ependyma make no contribution to SCI lesions by which they are not directly injured. Instead our findings suggest that ependymal cells may proliferate in particular to repair and seal off the central canal when it has been damaged. Our findings provide evidence that the proposal that ependyma are major contributors of neural stem cells after SCI is based on the use of (1) small radial SCI lesions that directly injure ependyma and exaggerate the contribution of ependymal progeny to the wound response, and (2) inappropriate claims regarding molecular markers used to identify putative astrocytes. Lastly, our findings strongly suggest that putative ependymal progenitors are not likely to be good candidates for endogenous manipulation or transplantation after SCI because of their restricted differentiation potential and limited migration capacity.

## Materials and methods

### Mice

All transgenic and non-transgenic mice used were derived from in house breeding colonies maintained on C57/BL6 backgrounds. All mice were between two and five months of age at the time of SCI. Transgenic mouse lines have been well-characterized previously: (1) *Foxj1*^*CreERT2*^ 29, (2) (td-tomato) reporter mice^30^. All mice were housed in a 12-hour light/dark cycle in a specific pathogen-free facility with controlled temperature and humidity and were allowed free access to food and water. All experiments were conducted according to protocols approved by the Animal Research Committee of the Office for Protection of Research Subjects at University of California Los Angeles.

### Tamoxifen

Tamoxifen was dissolved in corn oil and administered as subcutaneous injections. Uninjured mice were given tamoxifen, 75 mg/kg, once a day for 7 days followed by a 2-week drug washout period to avoid potential confounds of residual tamoxifen remaining during the first two weeks after delivery^53^, followed either by perfusion fixation for analysis of uninjured mice, or by SCI.

### Surgical procedures

All surgeries were performed under general anesthesia with isoflurane in oxygen-enriched air using an operating microscope (Zeiss, Oberkochen, Germany), and rodent stereotaxic apparatus (David Kopf, Tujunga, CA). Laminectomy of a single vertebra was performed and severe crush SCI were made at the level of T10 to expose the spinal cord. For severe spinal cord injury (SCI), No. 5 Dumont forceps (Fine Science Tools, Foster City, CA) without spacers and with a tip width of 0.5 mm were used to completely compress the entire spinal cord laterally from both sides for 5 seconds^7^,^20^. All animals received analgesic prior to wound closure and every 12 hours for at least 48 hours post-injury.

### BrdU

Bromodeoxyuridine (BrdU, Sigma) was dissolved in saline plus 0.007 N NaOH. For mice with 2 weeks survival times after SCI, BrdU was administered by intraperitoneal injections given twice daily at 100 mg/kg per injection, on days 2 through 7 after SCI, which is the well-established peak period of astrocyte proliferation after SCI in rodents^7^,^33^,^34^. For mice with 8 weeks survivals after SCI, BrdU was first administered as intraperitoneal injections given twice daily on days 2 through 7 after SCI, and thereafter once daily three times a week until tissue was harvested at 8 weeks after SCI.

### Histology and immunohistochemistry

After terminal anesthesia by barbiturate overdose, mice were perfused transcardially with a phosphate buffered saline rinse followed by either 4% paraformaldehyde or 10% formalin. Spinal cords were removed, post-fixed overnight, and cryoprotected in buffered 30% sucrose for 48 hours. Frozen sections (30 μm) were prepared using a cryostat microtome (Leica) and processed for immunofluorescence as described^7^,^12^,^13^. Primary antibodies were: rabbit anti-GFAP (1:1000; Dako, Carpinteria, CA); rat anti-GFAP (1:1000, Zymed Laboratories); sheep anti-BrdU (1:300, Maine Biotechnology Services, Portland, ME); rat anti-CD133 (1:200; Millipore, Temecula, CA); and rat anti-vimentin (clone # 280618; 1:150; Novus Biologicals, Littleton, CO). Fluorescence secondary antibodies were conjugated to: Alexa 488 (green) or Alexa 405 (blue) or to Cy3 (550, red) or Cy5 (649, far red) all from Jackson Immunoresearch Laboratories. Nuclear stain: 4′,6′-diamidino-2-phenylindole dihydrochloride (DAPI; 2 ng/ml; ThermoFisher). Sections were coverslipped using ProLong Gold anti-fade reagent (InVitrogen, Grand Island, NY). Sections were examined and photographed using deconvolution fluorescence microscopy and scanning confocal laser microscopy (Zeiss, Oberkochen, Germany).

### Quantification and statistical analyses

Cell counts were performed on 3-dimensional image stacks collected using x40 or x63 objectives to generate cells per volume values by investigators blinded to experimental groups and given only randomly assigned animal numbers. Areas or boxes counted are shown in schematics associated with specific graphs. At least one hundred cells were counted per animal for every discrete area-box evaluated. Means were generated per animal and these used to generate group means to compare for statistical analyses. Statistical evaluations were conducted either by t-tests for pair comparisons, and by ANOVA with post hoc, independent pair wise analysis as per Newman-Keuls for evaluations of repeated measures (Prism®, GraphPad, San Diego, CA). Group sizes were determined based on previous experience and post hoc power analysis was performed for all experiments using G * Power Software V 3.1.9.2^54^ as described previously^20^ and significance was only considered when power ≥0.8.

## Additional Information

**How to cite this article**: Ren, Y. *et al*. Ependymal cell contribution to scar formation after spinal cord injury is minimal, local and dependent on direct ependymal injury. *Sci. Rep.* **7**, 41122; doi: 10.1038/srep41122 (2017).

**Publisher's note:** Springer Nature remains neutral with regard to jurisdictional claims in published maps and institutional affiliations.
